# An effective modular approach for crowd counting in an image using convolutional neural networks

**DOI:** 10.1038/s41598-022-09685-w

**Published:** 2022-04-06

**Authors:** Naveed Ilyas, Zaheer Ahmad, Boreom Lee, Kiseon Kim

**Affiliations:** 1 Department of Biomedical Science and Engineering, Gwangju Institute of Science and Technology (GIST), Gwangju, 61005 Republic of Korea; 2 Department of Computer Science, COMSATS University Islamabad, Islamabad, Pakistan; 3 School of Electrical Engineering and Computer Science, Gwangju Institute of Science and Technology, Gwangju, 61005 Republic of Korea

**Keywords:** Engineering, Electrical and electronic engineering

## Abstract

Abrupt and continuous nature of scale variation in a crowded scene is a challenging task to enhance crowd counting accuracy in an image. Existing crowd counting techniques generally used multi-column or single-column dilated convolution to tackle scale variation due to perspective distortion. However, due to multi-column nature, they obtain identical features, whereas, the standard dilated convolution (SDC) with expanded receptive field size has sparse pixel sampling rate. Due to sparse nature of SDC, it is highly challenging to obtain relevant contextual information. Further, features at multiple scale are not extracted despite some inception-based model is not used (which is cost effective). To mitigate theses drawbacks in SDC, we therefore, propose a hierarchical dense dilated deep pyramid feature extraction through convolution neural network (CNN) for single image crowd counting (HDPF). It comprises of three modules: general feature extraction module (GFEM), deep pyramid feature extraction module (PFEM) and fusion module (FM). The GFEM is responsible to obtain task independent general features. Whereas, PFEM plays a vital role to obtain the relevant contextual information due to dense pixel sampling rate caused by densely connected dense stacked dilated convolutional modules (DSDCs). Further, due to dense connections among DSDCs, the final feature map acquires multi-scale information with expanded receptive field as compared to SDC. Due to dense pyramid nature, it is very effective to propagate the extracted feature from lower dilated convolutional layers (DCLs) to middle and higher DCLs, which result in better estimation accuracy. The FM is used to fuse the incoming features extracted by other modules. The proposed technique is tested through simulations on three well known datasets: Shanghaitech (Part-A), Shanghaitech (Part-B) and Venice. Results justify its relative effectiveness in terms of selected performance.

## Introduction

Crowd counting refers to count the number of individuals in an image or video^1,2^. The spatial distribution and density are two important indicators to understand the crowd dynamics^1^. Due to perspective distortion in images and videos, where some of the people are depicted by only few pixels, thus demands deeper investigation to develop a state of the art crowd counting (CC) technique. The performance of detection-based counting techniques increase in a sparse areas, however the counting accuracy degrades in high density scenes. In contrast, regression-based CC methods^3–5^ perform well in terms of counting accuracy in high density scenes. However, these methods face significant hurdles by relying on hand-crafted features extraction. Recently, CNN-based crowd counting methods^1^ have evolved to address the challenges faced by traditional CC techniques due to its ability to learn powerful features. For instance^1,6^, used CNN-based CC to obtain the estimated density. Similarly authors in Refs.^7,8^ proposed a multi-column network for density estimation. Different columns are explicitly designed for learning density variations across different feature resolutions. Despite high counting accuracy, the existing CNN-based CC techniques^1,6–8^ suffer from algorithmic weaknesses such as missing theory and reasoning.

Existing CNN-based crowd counting techniques enhance the counting accuracy by using well known networks such as multi-column, multi-tasking and dilated networks^1^. These networks have been widely used individually or in a combination with each other to increase the counting accuracy at the cost of different disadvantages. Firstly, multi-column networks generally obtain similar features, whereas, multi-scale contextual information with enhanced receptive field is obtained by using multi-column dilation network^9^. Some of the existing CNN-based networks used single column with varying dilation rates to obtain enhanced feature map resolution^7^. Besides advantages of dilated networks (single and multi-column), they still suffer from major short coming such as large training time, ineffectual branch structure, sparse pixel sampling rate, information loss and extraction of irrelevant information^9,10^. For example, authors in Refs.^7,8^ used multi-column architecture for density estimation by taking advantages of different receptive fields. The kernel size in each column is fixed thus handle a specific set of density (images) scenes. Further, multi-column architecture used in Refs.^7,8^ failed to obtain high CC accuracy due to learning similar types of features^10^. In addition, by averaging the density maps from each column’s reduces the resolution of estimated density map. Secondly, multi-tasking such as pre-classification, segmentation^11^ and density-aware networks^12,13^ are incorporated in counting algorithms which make the network more complex because the number of parameters increases, leading to excessive memory usage making it impossible to monitor scenes in real time^14–16^. Lastly, dilated convolution with single or multi-columns is vulnerable in term of information loss in high dilation rate layers^9,10^. Further, enhanced receptive field without multi-scaling results in extraction of inappropriate information^10^. In addition, the pixel sampling rate of receptive field is very sparse, which results in extraction of irrelevant contextual features^9,10^. Moreover, the task independent general features at lower layers with task specific features at higher layers are not fully utilized to enhance the counting accuracy^10^.

Based on the above observations, we propose a hierarchical dense dilated pyramid feature extraction network. Our model comprises of three modules, (i) general feature extraction module (GFEM), (ii) Pyramid Feature Extraction Module (PFEM) and (iii) fusion module (FM). The first modules obtains simple to complex deep features. The second module consists of multiple sub-modules of deep stacked dilated convolution (DSDC). The DSDC is an improved version of standard dilated convolution (SDC) which is used to reduce the information loss caused by SDC. Further, by using dense connections among dilated convolution, the pixel rate per feature map increases which results in extraction of relevant contextual information in large dilation rates. DSDCs are responsible for increasing the number of scales hence effective for multi-scale dense feature extraction without increasing the network complexity. The main contribution of our research are summarize as follows.

1. We design a deeper and denser hierarchical CNN-based CC network to obtain abrupt to continuous varying scale features. Densely organized DSDCs to aggregate the local to global information at final density map.
2. The proposed ensemble network of GFEM, PFEM, and FM improves the ability of the network to obtain large scale contextual information, handle the perspective distortion, expand the spatial sampling location and increase the number of scales.
3. The proposed aggregation-based approach of task independent and task specific features at higher layers from lower and middle-lower layers enhances the estimation accuracy.

## Related work

Rapid growth of CNN-based methods in classification, and segmentation tasks, the CNN-based techniques proved promising results in density estimation and CC. CNN-based CC methods face a lot of challenges such as perspective distortion, density level variation, non-uniform crowd distribution. To overcome these challenges, researchers play their role to develop a state of the art CC method.

Detection-based techniques for crowd counting utilize a moving-window detector to identify objects and count the number of people in an image^17^. Extraction of common features from appearance-based crowd images to count crowd, however they have limited recognition performance in dense crowded scenes. To overcome this issue, researchers used part-based methods to detect the specific body parts such as the head or the shoulder to count pedestrians^18,19^. However, these detection-based methods are only suitable for counting sparse crowds because they are affected by severe occlusions.

To address the problem faced by detection-based techniques, regression-based methods have been introduced for crowd counting. The main idea of regression-based methods is to learn a mapping from low-level features extracted from local image patches to the crowd count^20,21^. These extracted low-level features include edge, textures, foreground and gradient features such as local binary pattern, and histogram oriented gradients. Authors in Ref.^22^ proposed a new and accurate counting model based on YOLO_v3 to automatically and efficiently count dense steel pipes by images. To promote counting models-development and verification, a large-scale steel pipe image data set including various on-site conditions was constructed and publicly available. The proposed model was observed to be superior to the original YOLO_v3 detector in terms of average precision, mean absolute error, and root-mean-square error based on the steel pipe data set. Whereas authors in Ref.^23^ employs 11 well-known CNN models as the feature extractor of the YOLO_v2 for crack detection. The results confirm that a different feature extractor model of the YOLO_v2 network leads to a different detection results.

The regression approaches include linear regression^24^, piece-wise linear regression^25^, ridge regression^26^, and Gaussian process regression. These methods refine the previous detection-based ones, however they ignore spatial distribution information of crowds. To utilize spatial distribution information, the method proposed by Lempitsky and Zisserman^27^ regresses a density map rather than the crowd count. The method learns a linear mapping between local patch features and density maps, then estimates the total number of objects via integrating over the whole density map. Whereas, method proposed by Pham et al.^28^ learns a non-linear mapping between local patch features and density maps by using random forest.

Due to strong representation ability of CNN’s, a wide variety CNN-based crowd counting techniques have been proposed. Benefited from CNN’s strong ability to learn representations, a variety of CNN-based methods have recently been introduced in crowd counting. A pioneering work for CNN-based crowd counting proposed by Wang et al.^29^ used multiple convolutional layers to extract features and sent these features into a fully connected layer to estimate density in dense crowded environment. Another work done by authors in Ref.^30^ by fine-tuning the pre-trained network on specific scenes by selecting similar image patches from the training data. The main drawback is that the approach requires perspective information which is not always available. Due to variation in densities and appearance of a crowded image, authors in Refs.^7,8^ proposed a multi-column network for density estimation. Different columns are explicitly designed for learning density variations across different feature resolutions. Despite different sizes of filters, it is difficult for different columns to recognize varying density crowds, and this lack of recognition results in some ineffective branches. Sindagi and Patel^13^ proposed a multi-task framework to simultaneously predict density classification and generate the density map based on high-level prior information. Authors in Ref.^31^ proposed a method of fusing multi-scale density predictions of corresponding multi-scale inputs, while^32^ designed an aggregated multi-column dilated convolution network for perspective-free counting. However, none of these works consider local information. To avoid the issues of ineffective branches and expensive computation in previous multi-column networks, Li et al.^10^ introduced a deeper single-column-based dilated convolutional network called CSRNet. Reference^16^ developed an encoder decoder-based scale aggregation network for crowd counting.

## The proposed model

**Figure 1:**
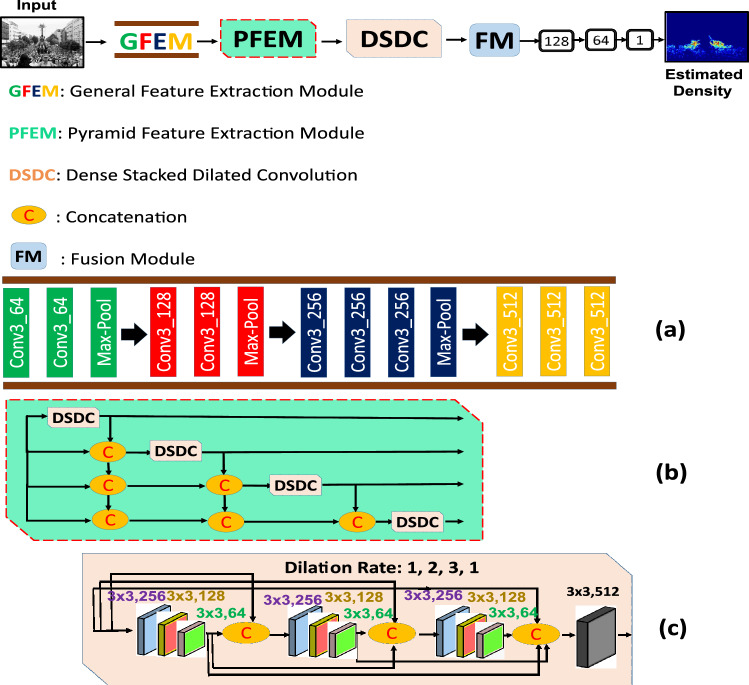
The overview of HDPF, A hierarchical dense dilated deep pyramid feature extraction CNN for single image crowd counting (top). The general feature extraction module **(a)**, the pyramid feature extraction module (PFEM) with four deep stacked dilated convolution (DSDC) blocks densely connected with each other **(b)**, while output is fused through fusion module (FM). The expansion of DSDC with multiple convolution layers densely connected with each other **(c)**.

The detailed architecture of the proposed technique is depicted in Fig. 1. Firstly, CC starts from ground truth density (GTD) estimation. Due to different density level, and varying head sizes in different CC datasets, different values of standard deviation ($\sigma$) are chosen for different dataset.

Secondly, our proposed technique employs GFEM which is used to obtain simple to complex deep features. Thirdly, our proposed network utilizes PFEM which enables the network to extract deep, multi-scale, and relevant information. Fourthly, multiple DSDCs in PFEM are densely connected with each other, thus enhancing the ability of the network to handle perspective distortion while increasing the number of scales and spatial sampling locations. We dedicate the following sections for HDPF, and HDPF outcomes, respectively.

### HDPF: a hierarchical dense dilated deep pyramid feature extraction through CNN for single image crowd counting

An end-to-end hierarchical dense dilated deep pyramid feature extraction CNN for Single Image Crowd Counting is depicted in Fig. 1. Our network with denser scale diversity is capable to cope with smooth to abrupt scale and density level variations. The proposed network employs three modules: GFEM, PFEM, and FM. The GFEM with smaller and same size of filters used to extract general feature (inspired from VGG-16^33^). This network is used to obtain simple to complex features. The PFEM with multiple DSDCs are densely deployed to obtain multi-scale, relevant contextual information while increasing the spatial sampling location (larger receptive field with multiple scales). Finally, the FM is used to fuse multiple inputs from different DSDCs.

#### General feature extraction module (GFEM)

Performance of CNN-based CC method usually increases on large training data. However, due to unavailability of training data, researchers^10,34^ used pre-trained model to reduce over-fitting. Authors in Ref.^35^ revealed that front-end of a network learn general features whereas, back-end responsible to obtain task specific features. Hence, we incorporate first ten layers of VGG-16^33^ in our proposed network. To understand the feature learning process in a deeper level, we divide ten layers in four groups depicted in Fig. 1 with different colors (green, red, dark blue, yellow). Convolutional layers are used in each group to maintain the spatial information with total of three down sampling layers (max pooling). In Fig. 1a, groups 1–2 (depicted by green and red colors) are responsible to learn very low level features such as curves, dots, and lines. Whereas, group 3 (dark blue color) is used to obtain complex features (like corners, edges) and group 4 (yellow color) is used to obtain the blobs. Hence, GFEM is very useful to learn simple to complex features, strong transfer learning capability, make it flexible to concatenate with any network.

#### Pyramid feature extraction module (PFEM)

Pedestrians in crowded scene usually contain different sizes due to multi-scale variation caused by perspective distortion. Where, extraction of irrelevant contextual information leads to reduced estimation accuracy. To address these challenges, the estimated feature map must be comprised of multi-scales information with expanded receptive fields. Therefore, inspired by the success of Refs.^36,37^, we built a PFEM by cascading several DSDCs with multi-dilation rate. We include four DSDCs in PFEM module that are densely connected with each other. The upper DSDC accepts output from lower ones, which results in larger and denser receptive field with multiple scales. In parallel mode, DSDCs accept inputs and their outputs are densely concatenated to obtain dense pixel and scale sampling rate.

**Table 1:** The architecture of HDPF.

Modules	Sub-modules	Channels	Filter size	Padding	Dilation	HADF
	Sub-M1	64	3 × 3	1	1	Conv3-64
						Conv3–64
						Max pooling
	Sub-M2	128	3 × 3	1	1	Conv3-128
						Conv3-128
						Max pooling
	Sub-M3	256	3 × 3	1	1	Conv3-256
						Conv3-256
						Max pooling
	Sub-M4	512	3 × 3	1	1	Conv3-512
						Conv3-512
						Conv3-512
	Sub-M5	512, 256, 128, 64	3 × 3	1	1	Conv3-512-1
						Conv3-256-1
						Conv3-128-1
						Conv3-64-1
	Sub-M6	576, 256, 128, 64	3 × 3	2	2	Conv3-576-2
						Conv3-256-2
						Conv3-128-2
						Conv3-64-2
	Sub-M7	640, 256, 128, 64	3 × 3	2	2	Conv3-640-2
						Conv3-256-2
						Conv3-128-2
						Conv3-64-2
	Sub-M8	640, 512	3 × 3	3	3	Conv3-640-3
						Conv3-512-3
Output		512, 128, 64, 1	3 × 3	1	1	Conv3-512-1
						Conv3-128-1
						Conv3-64-1
						Conv1-1-1

*Dense scale dilated convolution (DSDC)* The diverse scale variation across an image, perspective variation and density level variation are challenging issues in crowd counting. Therefore, multi-size filters, larger receptive field, dense pixel and scale sampling rate are useful to obtain state of the art estimated density with enhanced resolution density map. Inspired by the success of Ref.^37^, we propose two dimensional (i) cascaded (ii) parallel network. In cascading mode, the upper dilated convolutional layer (DCL) take the output of lower DCL to produce an efficient and larger receptive field. Whereas, in parallel mode, multiple DCL take the same input and their outputs are concatenated to obtain the output with multi-scale receptive field.

Due to continuous nature of scale variation in a crowded image, continuous and dense feature extraction is necessary to handle perspective distortions. We therefore, propose a dense scale dilated convolution module (DSDC), comprised of multiple DCL with varying dilation rates as shown in Table 1. Sub-Modules (Sub-M) in the Table 1 belongs to different modules. For example, Sub-M1—>Sub-M4 belongs to GFEM, whereas Sub-M5—>Sub-M8 belongs to PFEM, respectively. By using this setting, relevant contextual information is preserved with denser pixel sampling rate. The output of each DCL is connected with the input feature map, and all the outputs from lower DCL, and the concatenated feature map is fed into following DCLs. The final output of DSDC has a feature map with multi-dilation rate, multi-scale receptive field with larger relevant contextual information. In this way, the proposed network is used to obtain denser and larger pyramid feature by using only few DSDCs. The concatenation of multiple DCLs is carried as follows^37^.

1 $$\begin{aligned} Y_{l}=H_{K,d_{l}}([Y_{l-1},Y_{l-2},...,Y_{0}]), \end{aligned}$$

where $Y_{l}$ is the output of concatenated inputs, $d_{l}$ is the dilation rate of DCL (l), and $[Y_{l-1},...,Y_{0}]$ means the feature map formed by concatenating the outputs from previous DCLs. As compared to^38^, we stacked all the DCLs together, and connected them with dense connections. Due to this, our network obtained benefits: (i) denser feature pyramid, and (ii) larger receptive field (LRF). We will explain our proposed techniques in terms of these two advantages in the next section.

#### Fusion module

Multiple DSDCs are used to capture multi-scale, contextual and deep features information for estimated density map. Each DSDC is capable for obtaining intermediate density maps which have partial scale and relevant contextual information. Dense connections are used to combine partial information coming from multiple DSDCs. By doing this, the output of one DSDC has direct access to each layer of the subsequent DSDCs, resulting in a contiguous information pass. As compared to SDC, its scale diversity is further enlarged and suitable features for specific scenes are preserved adaptively during the flow process of information. Finally, the output from subsequent DSDCs (densely connected with each other) are aggregated by using fusion module (FM).

## HDPF outcomes

### Dense dilated deep pyramid feature extraction

HDPF is a hierarchical denser pyramid feature extraction network. The output receptive field in SDC increases with increasing dilation rate, however, it has sparse pixel sampling rate. Compared to SDC, as the dilation rate goes to higher side, the receptive field increases with multiple scales information and dense pixel sampling rate. So, denser network has the following features: (i) denser scale sampling rate (ii) dense pixel sampling with relevant information extraction (iii) better extraction of local and global information, and (iv) better scale diversity.

#### Denser scale sampling rate (DSSR)

The SDC is very effective to obtain contextual information with larger receptive filed size, however, it fails to obtain multi-scale information. To mitigate this problem in SDC, the concept of DSDC is introduced to stack multiple SDC layers with increasing dilation rate. In this way, the relevant contextual information with multi-scale information is obtained. The key idea is to use a dense connection among multiple DSDCs, hence lower dilation rate layers are incorporated with higher dilation rate layer to enrich the output feature map with multi-scale relevant contextual information.

Dilation is used to enhance the receptive field of a kernel. Suppose a SDC layer with dilation rate of *d* and kernel size *K*, the equivalent receptive field can be calculated by using (2).

2 $$\begin{aligned} R=(d-1)\times (K-1)+K. \end{aligned}$$

Let the convolution filter size of $3 \times 3$ with dilation rate ($d=3$), the corresponding receptive field is equivalent to $7\times 7$. By stacking two SDC layers larger receptive field with multiple scales can be obtained. Let us suppose two SDC layers with filter size $K_{1}$ and $K_{2}$, respectively, the output receptive field can be calculated by using (3).

3 $$\begin{aligned} K=K_{1}+K_{2}-1. \end{aligned}$$

**Figure 2:**
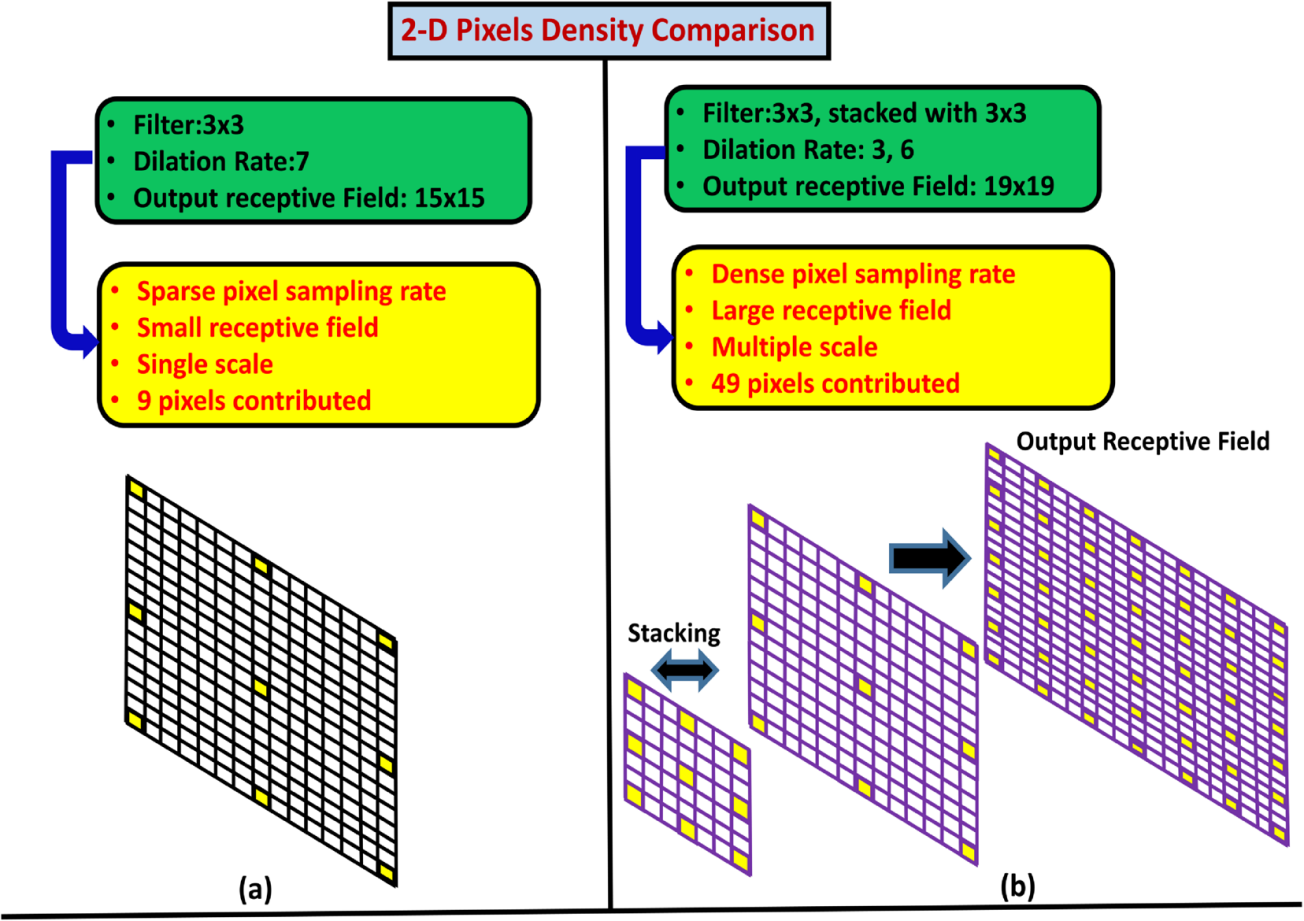
A comparison between SDC and DSDC in terms of pixels density and relevant contextual features, (**a**) SDC with dilation rate (7) results in smaller receptive field. (**b**) Stacking a dilated layer with smaller dilation rate (3) with larger dilation rate (6) make a denser sampling rate with larger receptive field.

Suppose we have a filter size of $3 \times 3$, dilation rate of 7, the output receptive field by applying SDC is equal to $15 \times 15$ as shown in Fig. 2a. By stacking two filters of the same size $3 \times 3$ with dilation rate of 3 and 6 respectively, we get an output receptive field of $19\times 19$ as shown in Fig. 2b. Hence, DSDC is very useful to obtain a larger receptive field and multi-scale information as compared to SDC.

#### Extraction of relevant contextual information

The SDC plays a vital role in expanding the receptive field size, hence obtaining the contextual information. However, as the dilation rate gets higher, despite a larger size of output receptive filed, the pixel rate gets sparse. The pixels of dilated kernel are very sparse, which result in extraction of irrelevant contextual information. To overcome this deficiency, the DSDCs are densely connected to increase the denser pixel sampling rate.

By using dense connections among multiple SDC layers, the output receptive field not only consists of multiple scales but also has dense pixel sampling rate with minimal relevant information loss as depicted in Fig. 2b. Let us understand the 2D pixels density comparison among the SDC and DSDC depicted in Fig. 2a,b. Where, Fig. 2a shows the SDC with filter size of $3 \times 3$, dilation rate of 7. The output receptive field is equivalent to $15 \times 15$. As depicted, the output receptive field has sparse pixel sampling rate, smaller receptive filed, single scale information and more importantly a very limited number of pixels contribution (9 pixels) for the next feature map. Fig. 2b depicts the DSDC with two filters of size $3\times 3$ stacked upon each other with dilation rate of 3 and 6 respectively. The equivalent output receptive field is equal to $19\times 19$. As compared to SDC on the left, the DSDC has denser pixel sampling rate, larger receptive field and has multiple scales. Further, increasing dilation rate layer by layer employs features from lower layer, improving the density estimation.

#### Aggregation of local and global information

The extraction and passing of information from lower and middle-lower layer to higher layers play a vital role in obtaining a higher segmentation accuracy^37^. And combination of task independent general features and task specific features are very useful to obtain the high counting accuracy^39^. Generally, the features extracted at lower layers are utilized locally^9,10^ and are not passed to higher layers, hence the result is low estimation accuracy. To solve this problem, the SDC layers are densely connected to subsequent layers, hence passing the locally obtained information to middle-higher and higher layers. In this way, local information at each layer is propagated with aggregation of global information in the final feature map. The final feature map has a larger receptive field with multi-scales and high dense pixel and sampling rate as shown in Fig. 2.

## Experiments

The experimental detail of proposed technique begin with network architecture to evaluation of proposed HDPF. We further divide this section into three sub-sections: implementation, ablation study, comparison with state of the art. Further, HDPF is evaluated on three well-known datasets namely ShanghaiTech (PART-A), ShanghaiTech (PART-B), and Venice dataset.

### Implementation details

#### Network configuration

The network configuration of HDPF is shown in Table 1. The proposed network is comprised of three main modules: GFEM, PFEM, and FM. These modules are further sub-divided into sub-modules as shown in Table 1. GFEM comprises of four sub-modules (Sub-M): Sub-M1 to Sub-M4, whereas, the PFEM consists of Sub-M5 to Sub-M8 respectively. GFEM is a modified form of VGG-16 network^33^ by using only ten layers to reduce the complexity.

To extract multi-scale, relevant contextual features, PFEM with FM is appended with GFEM. PFEM consists of smaller size filters with SDC layers with increasing dilation rates stacked upon each other. They are responsible to expand the receptive field size, increase the number of scales in resulting feature map, and enhance the dense pixel sampling rate. This enables the network to obtain the multi-scale information, extract relevant contextual information and enhance quality of density map.

#### Training details

The loss between estimated density and GTD is measured by using Euclidean distance. The loss function is given as follows:

4 $$\begin{aligned} L(\Theta )=\frac{1}{N}\sum _{i=1}^{N}\left\| Z(X_{i},\Theta )-G_{i} \right\| _{2}^{2}, \end{aligned}$$

where, $\Theta$ is a set of parameters. $X_{i}$ is the input and $G_{i}$ represents ground truth density. $Z(X_{i},\Theta )$ is the output density map for any input $X_{i}$, where N is the size of training images. To minimize the network complexity, only first 10 layers are used from pre-trained VGG-16 architecture^33^. The two most commonly used optimizer algorithms are Stochastic Gradient Descent (SGD) and Adam. One interesting and dominant argument about optimizers is that SGD better generalizes than Adam. Although literature reported that Adam converges faster, however, SGD generalizes better than Adam and thus results in improved final performance. The SGD with learning rate 1e−6 and momentum 0.9 is used in HDPF. We simulate the HDPF method by using PyTorch plateform^40^ with NVIDIA GeForce GTX 1070 with 8 GB memory.

#### Data preparation

Due to small number of training images, image augmentation is used to increase the number of training data. We cropped 9 patches from each image, whereas the size if each patch is 1/4 of original image. The first 4 patches are 4 quarter of an image, whereas rest of five patches are randomly cropped. We mirror the cropped image patches to further increases the training data. Whereas, whole images are used for test dataset (evaluation).

**Table 2:** Estimation errors on ShanghaiTech (Part-A), (Part-B), and Venice dataset.

Technique	Part-A		Part-B		Venice	
	MAE	MSE	MAE	MSE	MAE	MSE
Zhang et al.^30^	181.8	277.7	32.0	49.8	–	–
Marsden et al.^41^	126.5	173.5	23.8	33.1	–	–
Zhang et al.^7^	110.2	173.2	26.4	41.3	145.4	147.3
Sindagi et al.^13^	101.3	152.4	20.0	31.1	–	–
Sam et al.^8^	90.4	135.0	21.6	33.4	52.8	59.5
Zhang et al.^42^	86.8	139.2	16.2	25.8	–	–
Wang et al.^9^	83.7	124.5	17.9	32.4	–	–
Sindagi et al.^34^	73.6	106.4	20.1	30.1	–	–
Shen et al.^43^	75.7	102.7	17.2	27.4	–	–
Shi et al.^44^	73.5	112.3	18.7	26.0	–	–
Babu et al.^45^	72.5	118.2	13.6	21.1	–	–
Li et al.^46^	71.5	108.7	12.2	20.0	–	–
Wang et al.^47^	71.9	117.9	9.3	14.4	–	–
Ranjan et al.^48^	68.5	116.2	10.7	12.2	–	–
Li et al.^10^	68.2	115.0	10.0	16.0	35.8	50.0
Onoro et al.^49^	–	–	–	–	35.0	40.4
Liu et al.^50^	62.3	100.0	7.8	12.2	20.5	29.9
Proposed	69.9	106.9	9.4	15.9	16.3	23.9

### Comparison with existing algorithms

#### Evaluation metrics

The proposed technique is evaluated on three well known datasets such as ShanghaiTech (Part-A), ShanghaiTech (Part-B) and Venice. For evaluation purpose, we use mean absolute error (MAE) and mean square error (MSE) to calculate the loss or error count as given below:

5 $$\begin{aligned} MAE=\frac{1}{N}\sum _{i=1}^N|y_{i}-y^{'}_{i}|, \end{aligned}$$

6 $$\begin{aligned} MSE=\sqrt{\frac{1}{N}\sum _{i=1}^N(y_{i}-y^{'}_{i})^2}, \end{aligned}$$

where *N* denotes the number of images, $y_{i}$ is the estimated count and $y^{'}$ is the corresponding ground truth count.

#### Testing data

**Figure 3:**
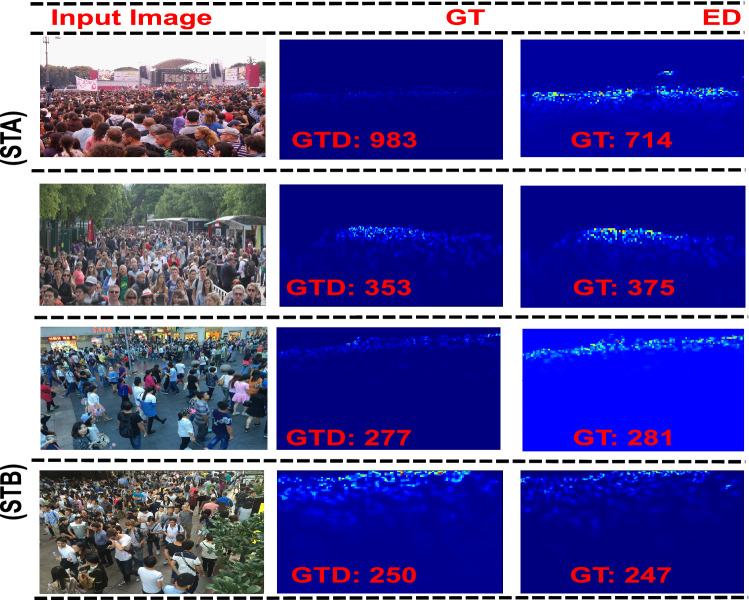
Visualization of ShanghaiTech Dataset (Part-A, Part-B)^7^, ground truth density, estimated density.

*ShanghaiTech (PART-A)* The ShanghaiTech (Part-A) dataset^7^ is a varying scale, densely populated, with diverse scenes and non-uniform density levels. We evaluate the HDPF on ShanghaiTech (PART-A) and compare it with state-of-the-art techniques^7–10,13,30,34,41–50^. Table  2 shows that performance in terms of MAE and MSE is competitive to the counterpart. The reason is the consideration of DSDC modules by aggregating the task specific relevant contextual features with task independent features extracted from lower layers. The qualitative results are shown in Fig.  3.

**Figure 4:**
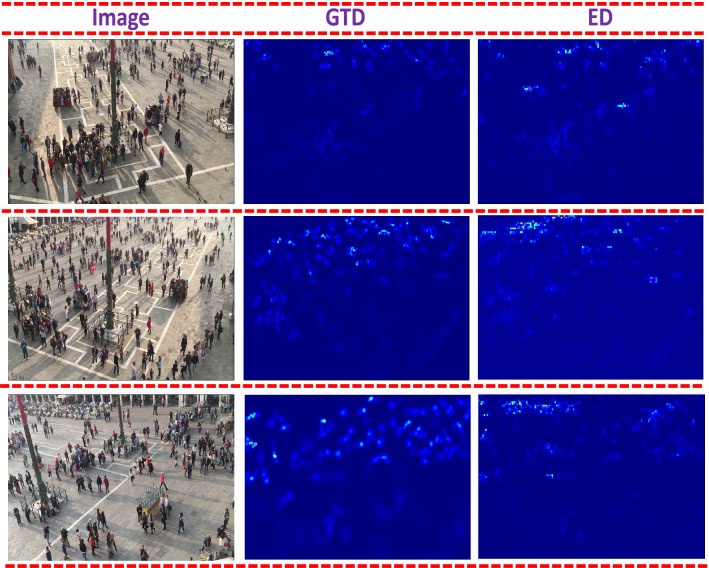
Visualization of Venice dataset, ground truth density, estimated density.

**Table 3:** A comparison between counting accuracy (MAE,MSE) and increasing number of DSDC’s. Whereas, (DSDC)[WD] stands for DSDC without dilation.

Option	No. of DSDCs	Venice dataset	
		MAE	MSE
GFEM	0	43.01	60.25
GFEM+4(DSDC)[WD]	04	23.83	34.59
GFEM+1(DSDC)	01	18.48	27.64
GFEM+2(DSDC)	02	18.82	25.81
GFEM+3(DSDC)	03	19.73	28.88
GFEM+4(DSDC)	04	**16.35**	**23.90**

Significance values are given in bold.

*ShanghaiTech (PART-B)* The ShanghaiTech (Part-B) dataset^7^ is a low density dataset compared to (Part-A) with varying scale and perspective. We compare HDPF with existing state of the art algorithms^7–10,13,30,34,41–50^ on ShanghaiTech datasets (Part-B). The performance of HDPF in terms of MAE and MSE is shown in Table  2. Further, the qualitative results are shown in Fig.  3.

*Venice dataset* The Venice dataset contains 167 images with fixed resolution of 1280 $\times$ 720. It is collected from Venice city with varying perspective. Sparse and non-uniform density levels make it a low density dataset. We compare HDPF with existing state-of-the-art techniques^7–10,13,30,34,41–50^. HDPF outperform the counterpart as shown in Table  2. The reason is consideration of SDC makes the dense connection which leads to high counting accuracy. Further, relevant contextual information aggregated at low level layers are propagated to higher layers thus enrich the final density map. Further, qualititative resultts are shown in Fig.  4.

### Architecture ablation

This subsection is dedicated to investigate the capability of each component of HDPF. We conduct all ablations on Venice dataset. To validate effectiveness of HDPF, we conduct experiments by adding components incrementally as shown in Table  3.

**Figure 5:**
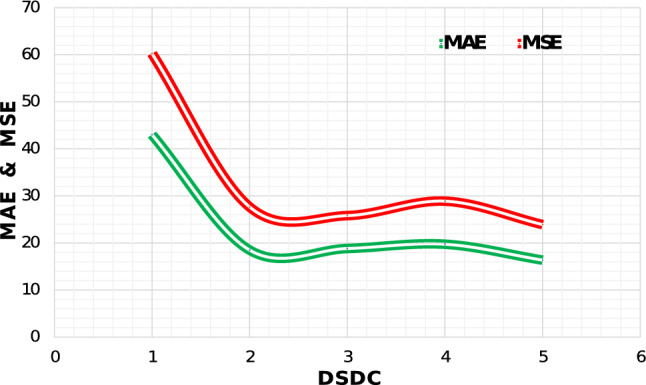
A comparison between counting accuracy (MAE, MSE) and increasing number of DSDCs.

The ablation study consists of five modules that are added sequentially.

- GFEM: GFEM is a VGG-16 based network.
- GFEM+4(DSDC)Without Dilation: GFEM is a VGG-16 based architecture with four DSDC modules and without any dilated convolution.
- GFEM+1(DSDC): GFEM with one DSDC and with dilated convolution.
- GFEM+2(DSDC): GFEM with two DSDC and with dilated convolution.
- GFEM+3(DSDC): GFEM with three DSDC and with dilated convolution.
- GFEM+4(DSDC): GFEM with four DSDC and with dilated convolution.

#### Effect of number of DSDC’s

We evaluate the proposed model by sequentially adding modules. Starting from GFEM which is based on first 10 layers of VGG-16 network. It achieves MAE of 43.01. Further, we use combination of GFEM with four DSDCs without any dilation rate and obtain MAE of 23.83. To find the optimal number of DSDCs, we perform experiment by sequentially adding the DSDCs. Results show that counting accuracy increases to a specific limit (number of DSDC equal to 4), however when we further added the number of DSDCs, the accuracy degrades sharply as shown in Fig. 5 (right). After finding an optimal number of DSDCs, we perform our experiment on different datasets to further analyze the effectiveness of our proposed network.

## Conclusion and future work

In this work, we proposed a novel architecture called a hierarchical dense dilated deep pyramid feature extraction through CNN for Single Image Crowd Counting that is trained in an end-to-end manner. Due to strong relevant feature aggregation property from lower and lower middle layer to higher layer, performance of the network is enhanced in terms of counting accuracy. Multi-scale feature extraction with expanded receptive field has improved the relative counting accuracy of HDPF. Further, the dense pixel sampling rate are useful to obtain relevant contextual feature extraction. In addition, our proposed network is capable of learning low to complex, deeper, multi-scale-aware, relevant contextual features. The combination of local and global features have further enhanced the counting accuracy. In future, we intend to use un-supervised learning with manifold approach to improve the counting accuracy further. Further, quality of density map is further enhanced by using Generative Adversarial Network (GAN), and we will consider GAN for future research work.
