# Author Correction: An effective modular approach for crowd counting in an image using convolutional neural networks

**DOI:** 10.1038/s41598-022-11280-y

**Published:** 2022-04-26

**Authors:** Naveed Ilyas, Zaheer Ahmad, Boreom Lee, Kiseon Kim

**Affiliations:** 1 Department of Biomedical Science and Engineering, Gwangju Institute of Science and Technology (GIST), Gwangju, 61005 Republic of Korea; 2 Department of Computer Science, COMSATS University Islamabad, Islamabad, Pakistan; 3 School of Electrical Engineering and Computer Science, Gwangju Institute of Science and Technology, Gwangju, 61005 Republic of Korea

Correction to: *Scientific Reports* 10.1038/s41598-022-09685-w, published online 06 April 2022

The original version of this Article contained an error in the Acknowledgments section.

“This work was jointly supported by the “GIST Research Institute (GRI) IIBR” grant funded by GIST in 202022 and the Korea Medical Device Development Fund Grant funded by the Korean government (the Ministry of Science and ICT; the Ministry of Trade, Industry and Energy; the Ministry of Health Welfare; the Ministry of Food and Drug Safety; NTIS Number: 9991006823). This work was also supported by the Technology Innovation Program (Industrial Strategic Technology Development Program-Development of Core Industrial Technology; Development of Navigation System Technologies of MicroNano Robots with Drugs for Brain Disease Therapy; the Ministry of Trade, Industry and Energy (MOTIE); NTIS Number: 20003822), South Korea.”

now reads:

“This work was jointly supported by the “GIST Research Institute (GRI) IIBR” grant funded by GIST in 2022 and the Korea Medical Device Development Fund Grant funded by the Korean government (the Ministry of Science and ICT; the Ministry of Trade, Industry and Energy; the Ministry of Health Welfare; the Ministry of Food and Drug Safety; NTIS Number: 9991006823). This work was also supported by the Technology Innovation Program (Industrial Strategic Technology Development Program-Development of Core Industrial Technology; Development of Navigation System Technologies of MicroNano Robots with Drugs for Brain Disease Therapy; the Ministry of Trade, Industry and Energy (MOTIE); NTIS Number: 20003822), South Korea.”

The original Article has been corrected.

